# TPEN—Advanced Metal Chelator: From Characterization to Biomedical Applications

**DOI:** 10.3390/molecules31142482

**Published:** 2026-07-16

**Authors:** Katarzyna Rydel-Ciszek

**Affiliations:** Department of Physical Chemistry, Faculty of Chemistry, Rzeszów University of Technology, Al. Powstańców Warszawy 6, 35-029 Rzeszów, Poland; kasiar@prz.edu.pl

**Keywords:** TPEN metal chelator, *N*-dentate ligand, TPEN analogues, TPEN complexes with d-, p-, and f-block metals

## Abstract

TPEN (*N*,*N*,*N*′,*N*′-tetrakis(2-pyridylmethyl)ethylenediamine) is a ligand containing six nitrogen donors. It is characterized by high structural flexibility, enabling the coordination of metals with various ionic radii and coordination numbers. It is an advanced metal chelator that demonstrates high selectivity, particularly towards “soft” and “medium” metal ions, and has a wide range of applications, from coordination chemistry, materials engineering, and nuclear energy to innovations in medicine. TPEN can cross cell membranes freely, which is important in cell biology. However, its presence is not neutral for healthy cells and can lead to apoptosis by depleting essential metals such as zinc, iron, and copper. Targeted delivery systems are therefore essential. This can be achieved, for example, by using nanoparticles that release TPEN upon ultrasound. This review systematizes the understanding of TPEN complexes. Methods for the coordination of various d-, p-, and f-block metals are presented, as well as the properties of these complexes, which are crucial to understanding the mechanisms of reaction with TPEN. This ligand may find applications both as a diagnostic tool (in sensors) and as a therapeutic tool (by inducing cancer cell death). This work also demonstrates the need to design new and more effective TPEN analogs that overcome problems with solubility and stability in acids.

## 1. Introduction

**Scheme 1:**
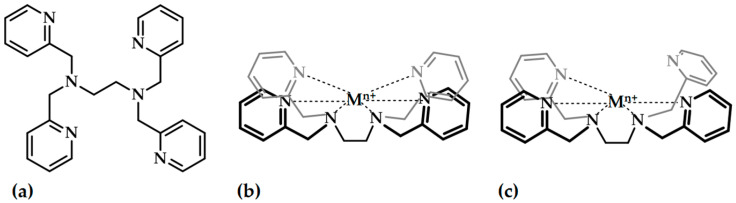
Structures of (**a**) the TPEN ligand and TPEN–metal complexes: (**b**) hexadentate and (**c**) pentadentate.

The TPEN ligand (*N*,*N*,*N*′,*N*′-tetrakis(2-pyridylmethyl)-1,2-ethylenediamine) [1] (Scheme 1a) has two types of nitrogen atoms: two aliphatic ones in the ethylenediamine bridge and four aromatic ones in the pyridyl rings, whose presence, in accordance with the theory of hard and soft acids and bases, classifies it as a medium base, showing a tendency to bind “soft” and “medium” (middle) metal ions [1,2]. It is highly soluble in water [2]. In aqueous solutions, the TPEN ligand undergoes gradual protonation. For protonated TPEN, four dissociation constants, K_1_ = 10^−7.2^ (pK_1_ = 7.2), K_2_ = 10^−4.8^ (pK_2_ = 4.8), K_3_ = 10^−3.3^ (pK_3_ = 3.3), and K_4_ = 10^−2.6^ (pK_4_ = 2.6), were presented [3]. These values are crucial to the stability of metal–TPEN complexes under various pH conditions. Protonation becomes negligible at pH above 2.5, allowing the [M(TPEN)]^n+^ form to dominate in solution and effectively coordinate the metal ions, where M = e.g., Cd(II) or Zn(II) [3]. It was presented [4,5] that the protonation of the pyridine groups in TPEN is because the pyridylmethyl substituent exerts a strong inductive effect that reduces the basicity of the aliphatic nitrogen atoms. Due to this reversal of basicity, protonation occurs primarily at the pyridyl groups, which are the main basic centers in TPEN. Even in strongly acidic solutions (pH ≈ 1), TPEN accepts as many protons as there are available pyridine groups, suggesting that the aliphatic centers remain unprotonated under these conditions. TPEN generates a stronger ligand field compared with simpler ligands (e.g., ethylenediamine), and due to its hexadentate nature, it forms very stable complexes with high stability constants [6] (Scheme 1b presents an example complex in which all six nitrogen atoms of the TPEN ligand are coordinated to the central metal ion). The presence of pyridine groups with low protonation constants reduces the tendency of the complexes formed with it to hydrolyze in aqueous solutions [7]. 

Due to its structure, TPEN is also called a tetrapyridyl, multidentate [8] ligand. It can act as a hexa-, penta- (Scheme 1b,c), or even tetra- or tridentate ligand, which allows the study of various coordination geometries within a single molecule [9]. It has a rather flexible structure. During the oxidation process, catalyzed by complexes of this ligand, a pyridyl arm can detach, which opens a free coordination site, allowing, for example, water molecules or OH^−^ ions to attach to the metal center [10].

As a high-density ligand, it ensures greater stability of the formed complexes, but an excessive number of coordinating arms can reduce their catalytic activity by blocking sites necessary for oxidant binding. The reduced efficiency of the catalyzed reactions is attributed to the presence of additional, unbound pyridine arms, which can compete with oxidant molecules for vacant coordination sites or cause steric hindrance, impeding the alkene’s access to the active site. In homogeneous catalysis, TPEN can contribute to extending the catalyst lifetime by preventing its decomposition into inactive metal salts, a problem encountered with ligands with fewer coordination sites [11]. It serves as a model compound in studies of pyridyl group exchange with solvent molecules, which is important in the design of stable catalysts and pharmaceuticals [9]. TPEN is a reference structure that can be modified (e.g., by methylation or the introduction of chiral centers) to improve the efficiency and regio- or enantioselectivity of catalytic processes [12]. It can also act as an effective chelating agent [13]. This ligand consists exclusively of C, H, and *N* atoms, so its combustion does not generate secondary solid waste, which is an advantage in the treatment of ecological waste, e.g., in the nuclear industry [14].

This review systematizes the knowledge about compounds formed with the TPEN ligand. It highlights directions for potential applications and suggests methods for modification to improve its properties, including its use as a drug.

## 2. TPEN Complexes with d-Block Metals

### 2.1. Iron Complexes

**Scheme 2:**
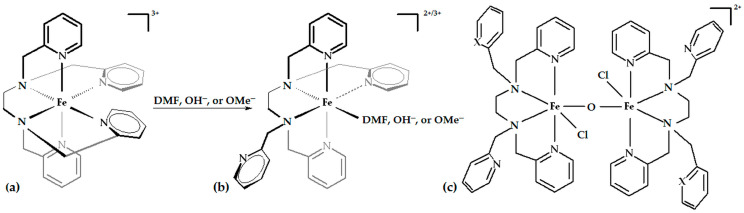
Structures of (**a**) the [Fe^III^(TPEN)]^3+^ complex, (**b**) the Fe(III)–TPEN complex in which one of the pyridyl arms was exchanged by a solvent molecule, and (**c**) μ-oxo iron complexes with TPEN (X = *N*) and Bn-TPEN (X = C) ligands [9].

In 1990, the synthesis of an iron(II) complex with TPEN, [Fe^II^(TPEN)](ClO_4_)_2_, with a stability constant of about 10^25^ was presented [1,15]. [Fe(TPEN)]^2+^ has been characterized by various methods and found to have a distorted octahedral geometry [16] (Table 1). A complex with Fe(III) ions, [Fe^III^(TPEN)][ClO_4_]_3_ (Scheme 2a), was also obtained [9], and X-ray crystallography showed that in the solid state, similarly to the complex with iron(II), it is characterized by a distorted octahedral structure around the metal center [9]. In methanolic and aqueous solutions, one of the pyridyl arms of the TPEN ligand is labile and undergoes exchange for a solvent molecule, while in DMF solution, this exchange occurs partially (Scheme 2b). Additionally, in solutions containing water, complexes of Fe(III) ions with TPEN and Bn-TPEN (Bn-TPEN pentadentate ligand–(*N*-benzyl-*N*,*N*′,*N*′-tris (2-pyridylmethyl)-ethylenediamine) are transformed into μ-oxo dimers, Fe-O-Fe, in which both ligands exhibit tetradentate character (Scheme 2c) [9]. This suggests that in complexes with iron, TPEN exhibits structural flexibility, allowing for a change in coordination number by decoordinating one of the pyridine arms, which can then act as a base in the second coordination sphere (Scheme 2a,b). For example, laser excitation causes changes in the structure of the [Fe^II^(TPEN)]^2+^ complex, resulting in asymmetric elongation of chemically unequal metal–ligand bonds. These changes result in a transition from the low-spin state (LS, S = 0), characteristic for the [Fe^III^(TPEN)]^3+^ complex [9], to the high-spin state (HS, S = 2) [8,12].

Transitions between the low- and high-spin states can also occur under the influence of temperature or light, and the degree of octahedral distortion increases with the increase in high-spin contribution [1]. For [Fe(TPEN)]^2+^, in acetonitrile, the transition from the low-spin to the high-spin state causes an increase in the solvation number and the formation of an internal solvation sphere of the complex. This transition results in a lengthening of the Fe–N bond and an increase in the angles between the pyridine rings. So, in the high-spin state, the complex structure becomes looser, allowing one solvent molecule, MeCN, to enter the internal cavity between the four pyridine rings. Therefore, the reactivity of [Fe(TPEN)]^2+^ is closely related to the spin state of the complex [17]. This spin-related lability of the system allows for a rapid reaction with oxidants [12]. The coordination of the metal ion in the second oxidation state (e.g., Fe(II)), which also depends largely on the reaction environment/solvents, causes a deformation from octahedral symmetry towards quasi-D_3h_ in which the pyridine planes are almost perpendicular to each other [8]. This deformation enables the formation of favorable interactions with the solvent and the synthesis of compounds with controllable excited states.

Iron(II) complexes with TPEN are much easier to isolate in solid form than their counterparts in the oxidation state III. Reaction with HOOH results in the formation of the adduct [Fe^III^(OOH)(HTPEN)]^3+^; this, however, is formed with a delay because the precursor [Fe^II^(TPEN)]^2+^ must first be oxidized to the +3 state. TPEN complexes with iron are referred to as “slow oxidants”; e.g., they oxidize very poorly cyclohexane to cyclohexanol and cyclohexanone [18]. The [Fe^II^(TPEN)]^2+^ complex can be used to activate hydrogen peroxide; reaction with HOOH results in the formation of the Fe^III^(OOH) form, which leads to Fe^IV^=O. The formation of these active catalyst species has been used in the hydroxylation of aromatic C-H bonds, e.g., in the oxidation of anisole to phenol or the corresponding methoxyphenols [12]. [Fe^IV^=O(TPEN)]^2+^ can also be obtained by reacting the iron(II) precursor with *meta*-chloroperoxybenzoic acid (*m*-CPBA), and it is stable for several hours at 0 °C but decomposes at room temperature, oxidizing the TPEN ligand itself. In the iron(IV)-oxo complex, one of the pyridyl arms likely remains uncoordinated, making it a pentadentate ligand in this particular form. Reactivity tests have shown the ability of this complex to oxidize hydrocarbons [19]. To exhibit activity, e.g., superoxide dismutase, the complexes must possess an easily exchangeable ligand or an unsaturated coordination structure, which allows for interaction with superoxide. [Fe(TPEN)]^2+^ can adopt two different structures depending on the type of accompanying anion; in the first, the iron is coordinated by six nitrogen atoms of the TPEN ligand (e.g., perchlorate or PF_6_^−^ anions), and in the second, one of the pyridyl groups is replaced by an anion (e.g., sulfate, SO_4_^2−^), creating a system in which the iron is coordinated by five TPEN nitrogens and one oxygen from the anion. The [Fe(TPEN)]^2+^ complex is stable towards oxygen—it does not undergo autoxidation for more than a month, which is attributed to its saturated coordination structure [20]. Therefore, [Fe(TPEN)]^2+^ can be used to protect cells from oxidative stress, for example, in *Escherichia coli* cells. TPEN and its complexes have been studied for their potential use in the treatment of diseases associated with free radical pathology, such as inflammation, ischemia-reperfusion injury, and cancer.

The [Fe(TPEN)]^2+^ complex can also react with NO, forming reversible nitrosyl bonds [21]. Therefore, these complexes can serve as a reference point in the search for more efficient NO “catchers” to control blood NO levels during sepsis. Overproduction of NO is dangerous and can lead to a rapid drop in blood pressure, and the binding of excess NO is intended to stabilize the patient’s condition. Thus, there is a need for biocompatible complexes that can not only bind NO but also transport it and catalyze its reactions [22].

**Scheme 3:**
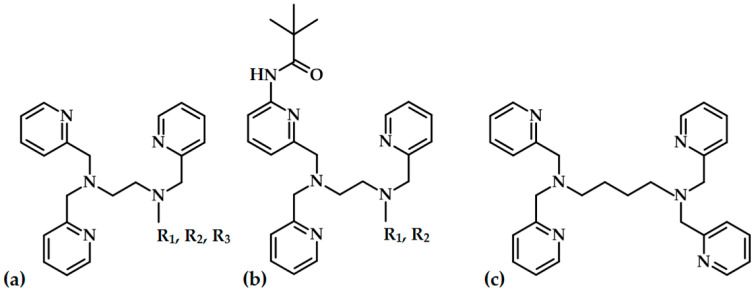
TPEN analogues, R_1_ = methyl (−CH_3_), R_2_ = benzyl (−C_7_H_9_), or R_3_ = dodecyl group (−C_12_H_25_). (**a**) Me-TPEN (R_1_), Bn-TPEN (R_2_) or C_12_-TPEN (R_3_) [23]. (**b**) 6-Amide-Me-TPEN (R_1_) or 6-Amide-Bn-TPEN (R_2_) [24]. (**c**) TPBN ligand.

Not only the TPEN ligand but also its analogues are used in the formation of various iron complexes. TPEN can be modified by selecting a substituent in one of the nitrogen atoms (e.g., a methyl, benzyl (Bn), or dodecyl group—an aliphatic chain; Scheme 3a) to give it specific properties [23].

This can be useful for the production of active, optically transparent NiFeO_x_H_y_ films, as electrocatalysts for solar water-splitting reactions, where the catalyst transparency is crucial to light access to the semiconductor. The NiFeO_x_H_y_ layer is formed from Ni(II) complexes with TPEN analogues (Scheme 3a). During the decomposition of nickel complexes on the electrode surface, Fe(III) ions present in solution are incorporated into the structure of the forming film, enabling the precise formation of the catalytic layer during the fabrication of transparent photoanodes [23]. Another application of TPEN analogues is the ability to regulate the stability and reactivity of iron complexes by modifying the ligand’s second coordination sphere—specifically through the introduction of an amide group and the change of substituents (e.g., to benzyl or methyl groups; Scheme 3b)—through which it is possible to influence the selectivity or efficiency of oxidation reactions catalyzed by iron(II) complexes [24].

Complexes with TPEN and Bn-TPEN ligands exhibit a number of similarities in iron binding. They can form complexes with iron(II) ions in a 1:1 ratio and are also capable of releasing iron from ferritin in the presence of reducing agents (ascorbate or dithiothreitol), making them useful in biological and biomimetic studies. The association constants (pFe^II^) are similar for TPEN (14.6) and Bn-TPEN (13.7), indicating that the loss of one coordinating group during the transition from a hexadentate to a pentadentate ligand does not drastically reduce iron binding strength. In terms of iron mobilization efficiency in the presence of ascorbate, the TPEN ligand proved to be the most effective, releasing 6.6% of total iron within 5 h, while Bn-TPEN released 4.2%. The Bn-TPEN ligand is primarily used to create stable iron(IV)-oxo species that mimic the activity of non-heme enzymes, while TPEN, despite its strong affinity for iron, is more often described as a specific zinc(II) chelator. TPEN also exhibits very strong binding to many divalent metals, with association constants (pMetal) of 18.0, 20.6, and 14.6 for Zn(II), Cu(II), and Fe(II), respectively. TPEN is capable of permeating cell membranes. It can be used to remove iron stores from ferritin, which is a method for depleting intracellular iron. It can also serve, like Bn-TPEN, as a model ligand for understanding the mechanisms of iron binding and enzyme reactivity [25].

Iron(II) complexes with *N*-pentadentate ligands have a labile water molecule in their coordination sphere, which facilitates their coordinative unsaturated state and access of oxygen to the metal center. In contrast, in the [Fe^II^(TPEN)]^2+^ complex, all six coordination sites around the iron center are occupied by the ligand’s nitrogen atoms, blocking access of O_2_ to the iron and preventing it from carrying out the redox reaction leading to enzyme oxidation. In the inhibition reaction of purified proteasome activity, iron complexes with Bn-TPEN are potent inhibitors; Bn-TPEN acts by activating oxygen and oxidizing the enzyme. This process leads to irreversible inactivation of the proteasome, which is important in the context of potential anticancer therapies. Unlike Bn-TPEN complexes, [Fe^II^(TPEN)]^2+^ does not exhibit time-dependent proteasome inactivation, but it is a good comparative ligand demonstrating that oxidative proteasome inactivation by iron complexes requires a free coordination site on the metal [26].

TPEN can be used for the selective detection of various divalent ions (Fe, Cu, Zn, Ni, Co, and Mn), and in samples containing iron ions, it acts selectively based on metal oxidation state. For instance, it enables the detection of Fe(II) ions from Fe(III) ions in unpurified biological samples, such as serum [2], highlighting its potential for medical applications in speciation analysis.

Table 1 presents the characteristics of TPEN complexes with iron ions and other d-block metal ions (discussed later), as well as the techniques used for their analysis.

**Table 1:** Metal complexes from the d-block with TPEN, with the complex anions and characteristics with references to the literature.

No.	Complex	Ligand Dentate in Complex	TPEN Geometry Type in Complexes	Complex Characterization Techniques
1.	[Fe^II^(TPEN)]^2+^; I^−^, BPh_4_^−^, CF_3_SO_3_^−^ [1]; ClO_4_^−^ [1,4,15,16]; PF_6_^−^ [1,18,19]	Hexadentate [1]	Octahedral [1,15,21]	Mössbauer, thermodynamic parameters, NMR [1], X-ray crystallography [1,15,16], UV–Vis [1,4,19,21], MS [19], CV (cyclic voltammetry) [12,18,27], DSC (Differential Scanning Calorimetry) analysis [16], IR [18], and EPR [21]
2.	[Fe^III^(TPEN)]^3+^; ClO_4_^−^ [9]	Hexadentate [9]	Distorted octahedral [9]	X-ray crystallography, UV–Vis, MS, and EPR [9]
3.	[Fe^IV^O(TPEN)]^2+^; PF_6_^−^ [19]	Pentadentate [19]	-	UV–Vis and MS [19]
4.	[Mn^II^(TPEN)(H_2_ O)]^2+^; ClO_4_^−^ [4,28]	Hexadentate [29]	Distorted capped trigonal prism [28,29] and pentagonal bipyramidal [5]	UV–Vis, MS [28], CV [27,28], X-ray crystallography, and EPR [28,29]
5.	[Co^II^(TPEN)]^2+^; ClO_4_^−^ [30]	Hexadentate [30]	Octahedral [30]	UV–Vis, CV, X-ray crystallography, and IR [30]
6.	[Co^III^(TPEN)]^3+^; ClO_4_^−^ [6,29]	Hexadentate [6,29]	Pseudo-octahedral [6,29]	UV–Vis [6] and X-ray crystallography [29]
7.	Cu^II^(TPEN)]^2+^; ClO_4_^−^ [4,31], PF_6_^−^ [5]	Pentadentate [5,31]	Distorted trigonal-bipyramidal and tetragonal-pyramidal [5]	UV–Vis [2,4,5,31,32], MS [5], CV [27,32], DFT [32], and X-ray crystallography [5,31]
8.	Cu^II^(TPEN)]^2+^; ClO_4_^−^ [33]	Hexadentate [33]	Distorted octahedral [33]	X-ray crystallography [33]
9.	[Cu^I^_2_(TPEN)]^2+^; BF_4_^−^ [34]	Tridentate [34]	Pseudo-tetrahedral [34]	NMR and X-ray crystallography [34]
10.	[Cu^II^_2_Cl_2_(TPEN)]^2+^; ClO_4_^−^ [31]	Tridentate [31]	Distorted square-planar [31]	UV–Vis, X-ray crystallography [31], and CV [35]
11.	[Zn^II^(TPEN)]^2+^; ClO_4_^−^ [4], PF_6_^−^ [5]	Hexadentate [5]	Distorted octahedral [5]	UV–Vis [2], X-ray crystallography, and NMR [5]
12.	[Ni^II^(TPEN)]^2+^; ClO_4_^−^ [4,10], PF_6_^−^ [36]	Hexadentate [36]	Octahedral [36]	UV–Vis [2,4], X-ray crystallography [27], CV [27,36], NMR, MS, and IR [36]
13.	[Re^IV^O(TPEN)]^2+^; ClO_4_^−^ [37]	Hexadentate [37]	Monocapped trigonal prism [37]	X-ray crystallography, CV, UV–Vis, and NMR [37]
14.	[Re^V^O(TPEN)]^3+^; ClO_4_^−^ [37]	Hexadentate [37]	Pentagonal bipyramid [37]	X-ray crystallography, CV [37], UV–Vis, and NMR [37,38]
15.	[Re^VII^(O)_3_ (TPENH)]^2+^; ClO_4_^−^ [39,40]	Tridentate [39]	Distorted octahedral [40]	X-ray crystallography [40] and NMR [38,40]

### 2.2. Complexes with Manganese, Cobalt, and Copper

**Scheme 4:**
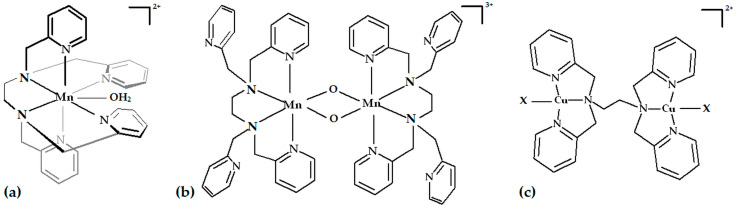
Structures of manganese [28] and copper [31] complexes with TPEN ligand: (**a**) [Mn(TPEN)(H_2_O)]^2+^, (**b**) [(TPEN)Mn^III^(µ-O)_2_Mn^IV^(TPEN)]^3+^, and (**c**) [Cu_2_X_2_(TPEN)]^2+^ when X = Cl, CO, or Br.

TPEN with d^5^ block metal ions, such as Mn(II), forms seven-coordinate structures. For example, there are a known single-core [Mn(TPEN)(H_2_O)](ClO_4_)_2_ complex (Scheme 4a) with a rare seven-coordinate geometry, which has been described as a “trigonal cap pyramid”; two-core [(TPEN)Mn(μ-OAc)Mn(TPEN)](ClO_4_)_3_∙2H_2_O, in which manganese ions are connected by an unusual acetate bridge; a compound with Mn(IV) ions–[(TPEN)Mn^IV^(µ-O)_2_(µ-OAc)Mn^IV^]^3+^; and a mixed-valence complex with a di-µ-oxo core [(TPEN)Mn^III^(µ-O)_2_Mn^IV^(TPEN)]^3+^, in which TPEN acts as a tetradentate ligand (Scheme 4b) [28]. 

With ions such as Co(III), this ligand forms stable six-coordinate structures [Co(TPEN)](ClO_4_)_3_ and has a pseudo-octahedral geometry [29]. In this complex, Co(III) ions are in a low-spin ground state and can be used to create Co(III) analogs for low-spin Fe(II) complexes [6]. The compound with Co(II) ions, [Co(TPEN)](ClO_4_)_2_∙3MeCN, has been used in the electrocatalytic oxidation of water. The cobalt(II) is also in an octahedral environment, surrounded by six nitrogen atoms of the ligand, which unfortunately hinders access by water molecules or OH^−^ groups to the cobalt metal center. This is the reason why cobalt complexes with TPEN are less effective than compounds with more flexible ligands, e.g., TPBN-*N*,*N*,*N*′,*N*′-tetrakis(2-pyridylmethyl)-1,4-butanediamine [30] (Scheme 3c). TPEN forms very stable complexes in a metal to ligand ratio of 1:1 with Mn^2+^ and Co^2+^ ions and acts as a hexadentate ligand (Table 1), while in reactions with Cu^2+^ and Zn^2+^, it exhibits a pentadentate character, which is confirmed by spectroscopic and thermodynamic data [4].

The [Cu(TPEN)]^2+^ complex is unexpectedly less stable, probably because it is penta-coordinated. One of the picolyl “arms” remains free. The geometry can vary from a tetragonal-pyramidal to a trigonal-bipyramidal structure depending on the anion (e.g., PF_6_^−^ vs. ClO_4_^−^) [5] (Table 1). Due to the electronic configuration of copper(II) and the specificity of TPEN, complexes with various geometries can be formed [32]. TPEN can be a pentadentate ligand, as is the case for the complex [Cu(TPEN)](ClO_4_)_2_, which is characterized by a distorted trigonal-bipyramidal geometry. Addition of chloride ions to this complex in methanol leads to the formation of a dinuclear copper(II) complex with formula [Cu_2_Cl_2_(TPEN)](ClO_4_)_2_∙H_2_O, in which the copper ions exhibit a distorted square-planar geometry, and TPEN is then a tridentate ligand for each of the copper ions [31] (Table 1; Scheme 4c). This ability to act as a bridge ligand and bind two copper ions simultaneously was also demonstrated for the dinuclear complex [Cu_2_(TPEN)](BF_4_)_2_ formed from a copper(I) salt [34]. Interestingly, upon saturation of a methanolic solution of this complex with carbon monoxide, a dicarbonyl adduct [Cu_2_(CO)_2_(TPEN)](BF_4_)_2_ is formed (Scheme 4c), and this process is reversible [34]. Each copper atom adopts a pseudo-tetrahedral geometry, binding to a CO molecule and three nitrogens of the ligand. TPEN can change shape (reorganize) depending on the presence of an additional ligand in the system; it can be used, for example, in reversible carbon monoxide capture and release processes [34]. A six-coordinate copper(II) complex with the TPEN ligand with formula [Cu(TPEN)](ClO_4_)_2_∙2/3H_2_O and a distorted octahedral geometry is also known.

Due to the rigidity of the ligand, it is structurally unstable and exhibits strong strains in the chelate rings, especially in the ethane-1,2-diamine plane [33]. This ligand was found to stabilize rare oxidation states, e.g., metal ions in low (e.g., Ni(I) and Cu(I)) or high (e.g., V(IV) and Ni(III)) valence states. In the case of complexes with copper(I) or vanadium(IV), it acts as a ligand that connects two metal centers [27]. Copper(I) complexes in the CuBr/TPEN system exist in an equilibrium state between the binuclear [Cu_2_Br_2_(TPEN)] (Scheme 4c) and mononuclear [CuBr(TPEN)] structures. In contrast, the structure of copper(II) complexes in the CuBr_2_/TPEN system is mononuclear [CuBr(TPEN)]Br, with one of the pyridyl nitrogens of the ligand remaining uncoordinated with the copper atom [35]. These reactions can be used in ATRP polymerization, where TPEN exhibits high activity; however, for catalysis to occur, the binuclear form must rearrange to a more active, mononuclear center. TPEN enables the preparation of polymers with a narrow molecular weight distribution, such as block copolymers, polymer brushes or hybrid materials, and its complexes are effective in the polymerization of methyl acrylate [35]. However, in the CuBr_2_/TPEN system, the halogen atom can coordinate with the “free” nitrogen atom of the ligand instead of the copper center, which can contribute to the loss of the catalyst deactivator form [41]. Multidentate TPEN has also been used in reactions of atom transfer radical addition (ATRA) [42]. In methanol, a typical solvent for ATRA reactions, the inactive form [Cu^II^(TPEN)]^2+^ predominates, while in acetone, the active [Cu^II^Br(TPEN)]^+^ complex is formed, in which Br ions displace one of the pyridine arms of the ligand [42]. The difference in activity of copper complexes results from the effect of the solvent on the ability to bind halide ions, and Cu(II) complexes with this ligand are particularly effective in less polar solvents (e.g., acetone). When used as a catalyst in ATRA reactions, it can be used to form new carbon–carbon bonds [42].

TPEN derivatives with polymerizable double bonds in the pyridine ring and their copolymers with *N*-isopropylacrylamide (NIPA) were used to obtain poly(TPEN–NIPA) gels. The resulting gels exhibited thermoresponsive properties, swelling at low temperatures and shrinking at higher temperatures. In the immobilized form in the NIPA gel, the stability of the complex or access to the active sites depends on the physical state of the polymer—complexation occurs efficiently at low temperatures when the polymer network is relaxed. The swollen gels were used to extract Cd(II) ions from aqueous solutions, whereas the shrunken gels produced almost no extraction. By introducing double-bond groups into the pyridine ring, TPEN can serve as a cross-linking agent in polymers, which allows for the imparting of new functionalities, such as thermoresponsivity [43].

TPEN, a potent copper chelator, has potential medical applications. It has been used in an innovative strategy against liver cancer. It was tested for copper-dependent cell death without the need for external copper delivery. This ligand, encapsulated in special “cancer-targeted nanoparticles”, was released by ultrasound and captured Cu(II) ions from enzymes such as superoxide dismutase, deactivating the cancer cell’s defense systems. The chelation process initiated a Fenton-like reaction (induced by glutathione), leading to the generation of large amounts of reactive oxygen species (ROS) and Cu^+^ ions. The resulting Cu^+^ ions bind to lipoylated proteins in the Krebs cycle, causing their aggregation and blocking mitochondrial metabolism, ultimately leading to cuproptosis [44]. Cell death induced by TPEN and ROS triggers the release of signaling molecules, and these signals stimulate dendritic cell maturation, leading to a strong infiltration of cytotoxic T lymphocytes into the tumor, transforming it from a “cold” tumor (with low immunogenicity) to a “hot” one that demonstrates a better response to treatment. Studies in animal models have shown that TPEN therapy is significantly more effective in inhibiting tumor growth than immunotherapy alone. By using nanoparticles targeting receptors found in excess in liver cancer cells, TPEN accumulates selectively in the tumor, limiting side effects in healthy tissues. TPEN is released from the carrier only after activation by ultrasound, allowing for precise control of the timing and location of drug administration. TPEN can be used to destroy liver cancer cells through a unique mechanism of cuproptosis, but it also unblocks the patient’s immune system, allowing it to fight cancer more effectively [44].

### 2.3. Zinc Complexes

TPEN is characterized by the versatility of forming complexes with d-block metal ions, including Zn(II) ions, to which it shows high affinity and with which it forms stable complexes [5]. TPEN is a potent, membrane-permeable chelator that is used in studies of oxidative stress, resulting in the release of Zn(II) ions in cells. TPEN contributes to the effective removal of free zinc(II) ions from the reaction environment or the cell interior [45]. In vitro, this ligand causes the disintegration of multimolecular complexes of “T-cell intracellular antigen-1” (TIA-1) into monomeric forms, and its activity demonstrates that Zn(II) interactions with TIA-1 are noncovalent and reversible. This property can be used to distinguish stress markers associated with, for example, unfavorable environmental conditions [45]. TPEN blocks the entry of TIA-1 into stress granules by depriving the protein of its physiological ligand, Zn(II) ions, released in response to cellular stress. In [45], oxidative stress was induced by sodium arsenite. Preincubation of cells with TPEN before arsenite administration significantly reduces the number of stress granules containing the TIA-1 protein. The action of TPEN is specific to TIA-1; this ligand does not have significant effect on the formation of stress granules. This suggests that zinc ions play a key role in TIA-1-dependent processes. By binding available Zn(II) ions, TPEN prevents TIA-1 from entering an aggregated state, which blocks its localization in stress granules [45].

TPEN, in reaction with zinc, is used to analyze the mechanisms of dopamine release and the functioning of glycine receptors, among others, in response to drugs and alcohol in addiction studies. It effectively blocks the increase in dopamine levels induced by alcohol [46]. This ligand can also, by binding zinc, reduce the activity of MAPK kinases and inhibit the production of proinflammatory chemokines in kidney cells, which allows for response control and is essential to understanding and potentially treating kidney diseases [47]. TPEN, therefore, plays a key role as a tool for monitoring metal-binding proteins. It allows for the proteome-wide mapping of metal–protein interactions. Proteins bound to the ligand have higher stability, making them more resistant to denaturation and remaining in solution. TPEN is used to induce destabilization of metal-binding proteins. By chelating zinc ions, metalloproteins lose their ligand (metal), which makes them susceptible to denaturation. This process has applications in proteomics and in the identification of target proteins [48].

TPEN is also used indirectly in cellular imaging studies of human melanoma cancer cells. Its proven high affinity for Zn^2+^ ions can be used to demonstrate that the observed reaction originates from this ion. For example, a sensor composed of 2,6-diformyl-4-methylphenol, characterized by low cytotoxicity, is used to image intracellular zinc concentrations in human melanoma cells (A375), producing light blue fluorescence. Importantly, Zn(II) ions displace Cd(II) ions from the complex with the sensor, creating a more stable structure, allowing for the detection of zinc even in the presence of cadmium. However, the addition of TPEN causes sensor decomplexation, the binding of zinc(II) ions, and the complete quenching of the fluorescence, confirming the reversibility and specificity of the process [49]. The chelation of Zn(II) ions can lead to an imbalance of reactive oxygen species [32]. Due to its ability to penetrate cell membranes, TPEN can be used as a specific zinc(II) chelator, inhibiting cell death and apoptosis induced by hypoxia and ischemia [50]. TPEN inhibits excessive potassium currents and partially restores the normal function of sodium channels, which are inactivated under ischemic conditions. Therefore, it is used to analyze the effect of zinc on the kinetics of voltage-gated potassium and sodium channels. Under pathological conditions, Zn^2+^ ions are released and accumulate in mitochondria, leading to their dysfunction and neuronal death. It has been suggested that binding Zn^2+^ ions interrupts the cascade of pathophysiological events and is a promising approach to counteracting neuronal loss after transient ischemia (e.g., during ischemic stroke or sudden cardiac arrest) [50]. TPEN helps maintain cell ionic balance by weakening potassium currents (the increase in which accompanies apoptosis) and partially inhibiting the negative impact of stress on the activity of sodium channels. The mechanism of its action allows us to understand the pathological cascade leading to brain damage and to assess the effectiveness of new neuroprotective substances [50]. TPEN effectively reduces intracellular free-zinc levels, which is used to simulate zinc deficiency in vitro [51]. This ligand is also used in cell death studies due to its ability to induce apoptosis to a much greater extent than milder extracellular chelators (such as diethylenetriaminepentaacetic acid) [52]. During HIV viremia, monocytes show a significant increase in the expression of genes involved in zinc metabolism. High zinc levels directly protect monocytes from cell death. TPEN has been used as a tool to reverse resistance to apoptosis. It has been investigated whether monocyte resistance to cell death is zinc-dependent. Through zinc chelation, TPEN effectively restored cell sensitivity to apoptosis to the level observed in uninfected individuals. Monocytes from HIV-viremic patients have been shown to have a higher threshold for the chelation response. Presumably, reducing HIV-induced zinc levels (for which TPEN serves as a model) could help eliminate viral reservoirs by sensitizing them to cell death [53].

TPEN has also been used in studies against the toxic effects of *Echis carinatus* snake venom. The main toxins responsible for local tissue damage are snake venom metalloproteases, which require Zn(II) ions for their catalytic activity. Studies have shown that TPEN inhibits the activity of snake venom metalloproteases, making it a potential first aid therapeutic agent in the treatment of snake bites. TPEN demonstrates high selectivity for zinc and does not disrupt calcium(II) ion homeostasis, which is crucial to avoiding interference with physiological processes such as blood coagulation. Therefore, it can be used as a tool for regulating the activity of zinc-dependent metalloproteases, which is important in research on diseases such as cancer and arthritis [54].

**Figure 1:**
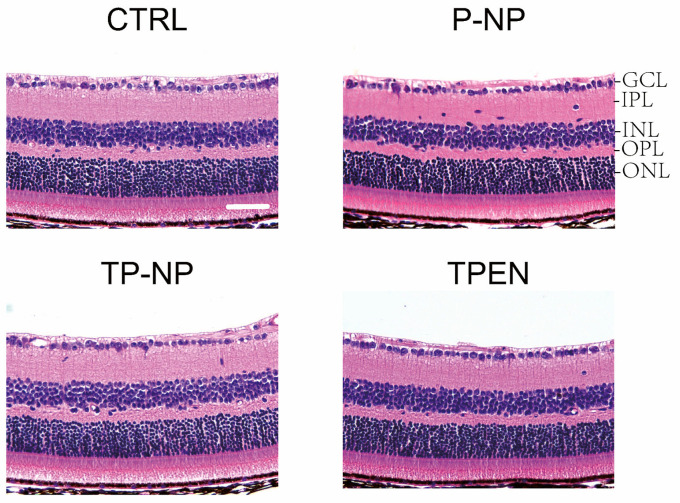
The effect of TPEN on the histological structure of the retina in vivo; GCL—ganglion cell layer; IPL—inner plexiform layer; INL—inner nuclear layer; OPL—outer plexiform layer; ONL—outer nuclear layer [55]. https://doi.org/10.1016/j.mtbio.2025.101670 (accessed on 18 May 2026). No changes were made to the Figure. This article is available under the terms of the Creative Commons Attribution License (CC BY); https://creativecommons.org/licenses/by/4.0/ (accessed on 18 May 2026).

A topic of interest is the development of an innovative TPEN delivery system for the treatment of glaucoma and other optic neuropathies. Biodegradable nanoparticles based on a polymer of polylactic acid–hydroxyacetic acid, coated with polydopamine, were developed to encapsulate and release TPEN. This system addresses the low bioavailability and hydrophobicity of TPEN itself, enabling its sustained release. Studies conducted in animal models (optic nerve crush injury and acute ocular hypertension) have shown that TPEN nanoparticles significantly increase the survival of retinal cell and support axonal regeneration. The nanoparticles demonstrated greater efficacy in chelating mobile zinc(II) in the retina after injury, which is crucial to reducing toxic zinc levels, as excess zinc initiates neuronal death. In vitro and in vivo tests confirmed that the developed nanoparticles are safe for ocular structures and do not cause histological damage to the retina or significant cellular toxicity at therapeutic doses. TPEN-modified nanoparticles represent a promising therapeutic strategy in preclinical studies for the treatment of glaucoma and other neurodegenerative diseases of the visual system [55]. The results of hematoxylin and eosin staining presented in Figure 1 showed that TPEN administration did not cause visible damage to retinal structures. Measurements of the thickness of individual layers showed that TPEN did not negatively affect the thickness of individual retinal layers—the results did not show statistically significant differences compared with the control group. The presented studies indicate that TPEN used in therapeutic doses is safe for the eye; it is a necessary condition for its potential clinical use [55].

**Figure 2:**
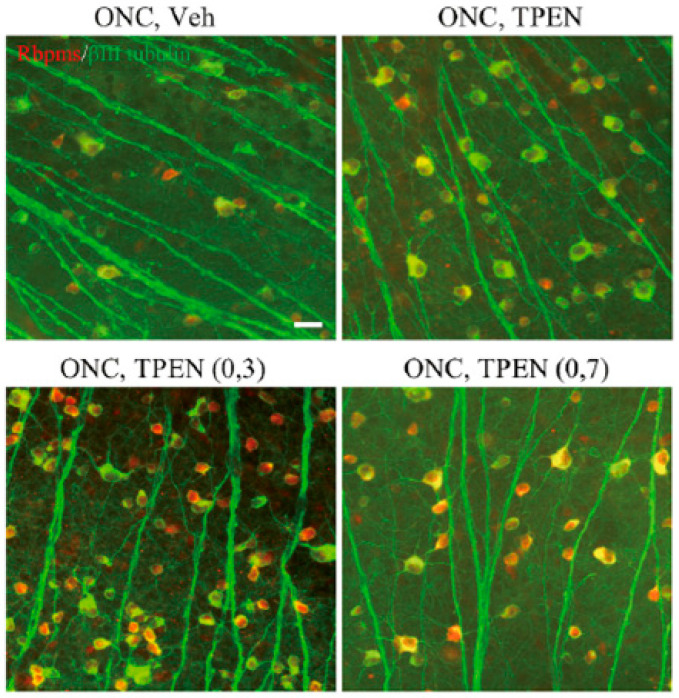
Effect of TPEN solution injection on neuronal protection and nerve regeneration after optic nerve crush (ONC) injury [55]. https://doi.org/10.1016/j.mtbio.2025.101670 (accessed on 18 May 2026). No changes were made to the Figure. This article is available under the terms of the Creative Commons Attribution License (CC BY); https://creativecommons.org/licenses/by/4.0/ (accessed on 18 May 2026).

In addition, it has been shown that after a single intravitreal injection, TPEN concentrations in retinal tissue decrease rapidly (Figure 2). An additional injection on the day after injury [TPEN (0,3)] significantly increased the number of surviving retinal ganglion cells compared with a single injection. It was found that administering a second dose of TPEN on day 7 [TPEN (0,7)] did not produce any significant improvement compared to a single injection. Increasing the frequency of injections of the TPEN solution improves the therapeutic effects, but the timing of the second dose is crucial. The improved results of the TPEN (0,3) group suggest a limited time window in which neurons can respond effectively to the zinc chelator. This information is important for the development of nanoparticles that provide sustained drug release, eliminating the need for multiple injections [55].

**Scheme 5:**
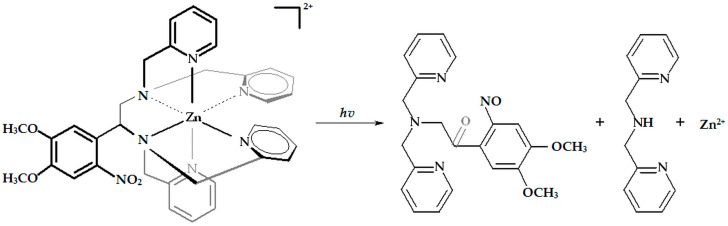
Photochemically induced release of zinc ions from the [Zn(ZinCleav-2)]^2+^ complex [57].

TPEN is also used in studies of intracellular calcium concentration using the fluorescent indicator Fura-2. Its use allows for the real-time monitoring of intracellular calcium changes in biological samples, such as neurons, without the risk of contamination by Zn(II) signal [56]. Another application of TPEN-like ligands is the construction of a photocage for Zn(II) ions (Scheme 5), enabling the controlled release of metals under the influence of light. Due to their high affinity, complexes based on the TPEN structure are able to retain metal ions even in the presence of cellular chelators, which is crucial to their stability against photoactivation [57]. TPEN derivatives can be used to precisely buffer zinc concentration at levels typical of the interior of cells [57]. TPEN interacts with the so-called zinc proteome and zinc sensors such as 6-methoxy-8-*p*-toluenesulfonamidoquinoline and Zinquin. TPEN can exchange with the sensor in a protein adduct, forming a new ternary adduct in which the metal is simultaneously bound by the protein and the TPEN fragment. It can quench the fluorescence of sensors not only by removing zinc from their complexes but also by extracting zinc ions directly from the proteome proteins and forming its own TPEN–zinc–protein adducts. TPEN is a promiscuous ligand that also influences the distribution of other metals, such as iron and copper, and its cellular toxicity results directly from its chelating properties [58]. TPEN is also used as a reference point for determining the binding constants of new sensors and for assessing their selectivity. It is used in ligand exchange reactions and in competition assays to determine the affinity of new fluorescent sensors for metal ions [59].

However, the use of TPEN as a zinc chelator may have several drawbacks and limitations. TPEN exhibits dose-dependent cellular toxicity. Although it is commonly used for zinc removal, it can disrupt other metabolic pathways, potentially leading to indirect effects on mitochondrial function that are not solely due to zinc deficiency. The TPEN-induced zinc deficiency model is an in vitro model of acute stress and, therefore, may not fully reflect the nature of chronic, systemic, and tightly regulated zinc deficiency observed in clinical patients. TPEN acts through rapid chelation in a cell culture model; caution should be exercised when translating the obtained results to in vivo models or directly to clinical practice. TPEN induces apoptosis, but the presence of the ATF3 protein (activating transcription factor 3) can limit this process by stabilizing mitochondrial function, preventing excessive mitochondrial degradation, and minimizing the negative effects of this chelator [60].

**Scheme 6:**
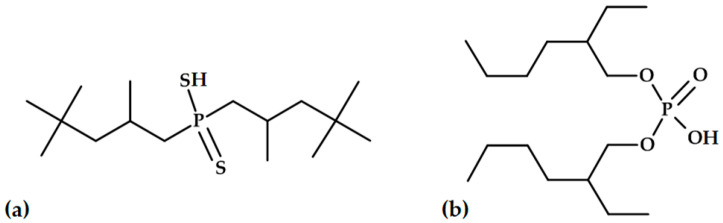
Structures of (**a**) Cyanex301 [bis(2,4,4-trimethylpentyl)dithiophosphinic acid] and (**b**) D2EHPA (di(2-ethylhexyl)phosphoric acid).

TPEN acts as a strong sequestering agent for Zn(II) ions, effectively blocking their extraction [3,61]. Separation of Cd(II) and Zn(II) ions is based on the formation of complexes of these metals with TPEN in the aqueous phase, which are then extracted into the organic phase. TPEN synergizes the extraction of cadmium while simultaneously sequestering the extraction of zinc, which allows for an increase in the separation coefficient of these metals compared with the system without TPEN. Therefore, this ligand serves as a means of promoting the extraction of soft metals (e.g., cadmium) using very small amounts [3]. The addition of another extractant—Cyanex301 [bis(2,4,4-trimethylpentyl)dithiophosphinic acid]—and an aqueous solution of TPEN to solutions containing Cd(II) and Zn(II) ions allows for a significant increase in the Cd/Zn separation coefficient. Adding this ligand in equimolar amounts to the heavy metals causes a drastic reduction in the level of zinc extraction, while the level of cadmium (a soft and toxic metal) extraction remains almost unchanged. This can be used for the effective recovery of heavy metals from wastewater and waste solutions [61]. TPEN added to the aqueous phase acts as an effective sequestering agent for zinc, forms a stable and extraction-resistant complex with Zn(II) in the aqueous phase while at the same time having a negligible effect on cadmium extraction, which, as a “soft” metal, exhibits a strong affinity for sulfur donors in Cyanex301 (Scheme 6a). As a result, it enables to achieve a high cadmium separation coefficient, which allows for the separation of soft from hard metals (e.g., separation of Cd from Zn) [62]. The combination of TPEN and the acidic extractant D2EHPA (di(2-ethylhexyl)phosphoric acid) (Scheme 6b) also allows for the selective separation of americium(III) from lanthanides(III) [63]. This ligand is used as a selective extractant, especially in synergistic systems with organic acids (e.g., D2EHPA or carboxylic acids), which allows for operation at lower pH. 

### 2.4. Complexes with Other d-Block Metals

**Scheme 7:**
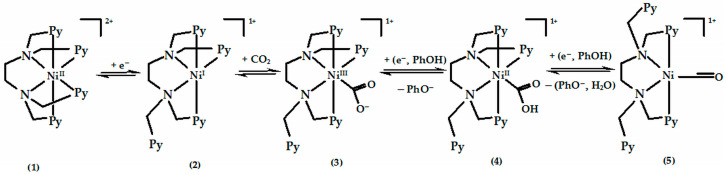
Putative mechanism for the electrochemical reduction of carbon dioxide. Compounds: (**1**) [Ni^II^(TPEN)]^2+^, (**2**) [Ni^I^(TPEN)]^+^, (**3**) [Ni^III^(TPEN)(CO_2_)]^+^, (**4**) [Ni^II^(TPEN)(CO_2_H)]^+^, and (**5**) [Ni(TPEN)(CO)]^+^ [36].

TPEN with transition metal and heavy metal ions forms predominantly cationic complexes with a 1:1 composition and a charge of 2+, which are characterized by high thermodynamic stability and kinetic inactivity [2]. The TPEN complex with Ni(II) adopts an octahedral geometry (Table 1), but the flexibility of the skeleton allows for easy adaptation to structural changes induced by redox processes, which favors the stability of the formed complexes over a wide potential range. For example, the complex [Ni(TPEN)](PF_6_)_2_, when reduced to Ni(I), readily reacts with CO_2_. One of the pyridyl arms of the ligand must dissociate, changing the coordination mode to pentadentate. Scheme 7 presents the mechanism of the electrochemical reduction of CO_2_ to CO and the catalyst degradation pathway that leads to the formation of the toxic Ni(CO)_4_. The vacant coordination site in complex (**2**) allows for the addition of a CO_2_ molecule. A nickel(III) complex (**3**) is formed, which is a key step in substrate activation. Depending on the environmental conditions, the process can proceed in two ways: under protic conditions (Scheme 7; with the addition of phenol, PhOH), complex (**3**) undergoes further reduction and protonation, resulting in compound (**4**), which, as a result of further protonation and reduction leading to the breaking of the C–O bond and the release of a water molecule (H_2_O), forms complex (**5**); and aprotic conditions (without a proton source), in which a reductive disproportionation reaction of two CO_2_ molecules occurs. This process results in the formation of carbonate (CO_3_^2−^) and, directly, complex (**5**). Complex (**5**) is not stable under reducing conditions. Further reduction and the addition of CO/CO_2_ molecules lead to rapid decomposition of the catalyst. Despite the high efficiency of CO_2_ activation, the [Ni(TPEN)]^2+^ complex requires structural modifications or the use of CO scavengers to prevent degradation and enable practical applications in catalysis [36].

Nickel compounds with TPEN exhibit a pH-independent Ni^III^/Ni^II^ oxidation process and a pH-dependent Ni^V^/N^iIII^ process associated with proton and electron transfer. The [Ni(TPEN)](ClO_4_)_2_∙0.5CH_3_COCH_3_ complex at pH 8.0 acts as a homogeneous molecular catalyst, while in a more alkaline environment (pH ≥ 9.0), it degrades, forming an active nickel oxide (NiO_x_) deposit on the electrode, which is used for oxygen production in the water oxidation process [10]. Additionally (as mentioned in Section 2.1), Ni(II) complexes with TPEN derivatives can be used to obtain transparent NiFeO_x_H_y_ films [23].

Some marine organisms, such as tunicates, accumulate high concentrations of both vanadium and sulfate in their blood cells. TPEN can be used to study the mechanisms of vanadium accumulation in marine organisms and to simulate environments with high acidity and high sulfate concentrations. The [V(SO_4_)(TPEN)]^+^ complex adopts a rare pentagonal bipyramidal geometry and is stable in aqueous solutions. The presence of such a large ligand in the vanadium(III) coordination sphere effectively inhibits the formation of stable μ-oxo dimers, which typically dominate the aqueous chemistry of this metal [64].

**Scheme 8:**
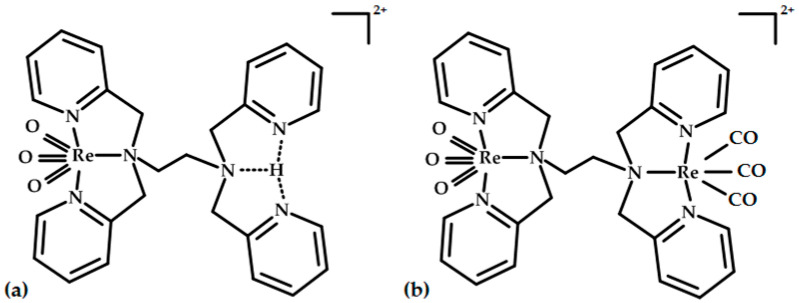
Structures of (**a**) [Re^VII^(O)_3_(TPENH)]^2+^ [39] and (**b**) [Re^VII^(O)_3_(TPEN)Re^I^(CO)_3_]^2+^ [40] complexes.

TPEN can be used as a so-called “complex ligand,” in which half of the ligand remains uncoordinated, allowing it to act as a ligand for other metal centers. Its structure allows it to be viewed as a bis(2-pyridylmethyl)amine derivative with a “giant substituent,” making it useful in the construction of multifunctional metal complexes (see, e.g., Scheme 8). This behavior was observed in mixed metal complexes, e.g., (Re^VII^–Fe^III^ and Re^VII^–Cu^II^), which demonstrated the ability to combine metal ions with extremely different oxidation states in a single molecule [39]. 

**Scheme 9:**
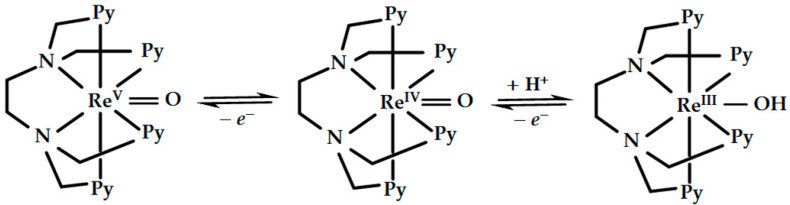
Structures of oxo-rhenium complexes [37].

With rhenium, TPEN can form seven-coordinate mononuclear oxo-rhenium complexes: [Re^IV^O(TPEN)](ClO_4_)_2_ and [Re^V^O(TPEN)](ClO_4_)_3_ (Table 1; Scheme 9). TPEN favors full coordination with a single metal ion, which allows for the isolation of rare coordination states, such as the seven-coordinated rhenium(IV) and (V) species (Scheme 9) [37].

It is a versatile framework for the formation of diverse complexes in a wide range of oxidation states (from Re(I) to Re(VII)). A rare, tridentate coordination mode of TPEN was found in the mononuclear complex with Re(VII) with formula [Re^VII^O_3_(TPEN)](ClO_4_)_2_ (Scheme 8a), in which one half of the ligand is bound to the metal center to which three oxygen atoms are attached, while the other half remains free (dangling arm). Such complexes with a partially coordinated ligand can serve as new building blocks that themselves behave as ligands due to the presence of free donor atoms. This feature of TPEN was used to create a heterobimetallic complex containing two rhenium centers in extremely different charges (+7 and +1), in which TPEN can act as a bridging ligand [40]; Scheme 8b. The rhenium(V)–[Re^V^O(TPEN)]^3+^ complex can spontaneously split in the presence of water into two other compounds with different oxidation states: rhenium(IV) and rhenium(VII). The reaction occurs in a 2:1 ratio (two molecules of the Re(IV) complex are formed for each molecule of Re(VII)) and is initiated by water molecules [38]. TPEN can be used to modify the surface of materials such as graphite oxide (GO) or silica (SiO_2_) by introducing reactive amine groups. This was utilized in the complex formed by anchoring the precursor ruthenium compound [RuCl_2_(PPh_3_)_3_] to the amine groups of the TPEN ligand immobilized on GO, which allowed for the preparation of new organic–inorganic materials with an ordered structure that can be used as nanoreactors. These compounds have been used, for example, in the chemoselective hydrogenation of cinnamaldehyde to cinnamyl alcohol [65].

## 3. TPEN Complexes with p- and f-Block Metals

### 3.1. Complexes with p-Block Metals

TPEN can bind to a metal in a hypodentate manner. In the presence of gallium(III), this ligand often utilizes only four of its six donor sites, leaving the two pyridine rings “dangling” (unbound): [Ga(TPEN)Cl_2_]Cl [11]. The pyridyl groups present in TPEN lower the protonation constants of the ligand. This allows for the formation of stable complexes with Lewis acidic metal ions, such as indium(III), and thus protects selected ions from hydrolysis in the aqueous environment of the body. This property of the ligand can be exploited in the design of compounds for biomedical applications by creating carriers that prevent the release of toxic metals in tissues (for example, in radiochemistry and in diagnostic imaging, including ^111^In and ^113^In isotopes [13]). Indium(III) exhibits affinity for nitrogen ligands in aqueous solutions. With TPEN, it forms a seven-coordinate complex [In(TPEN)(MeCOO)](ClO_4_)_2_∙0.5H_2_O, in which there are six bonds with the ligand nitrogen atoms (In–*N*), the seventh being occupied by the acetate group [13]. TPEN demonstrates the ability to form strong complexes at low pH and enables the study of the chemical properties of other metal ions, such as Bi(III) and Pb(II) [66]. With Tl(III) ions, the complex [Tl(TPEN)(NO_3_)](ClO_4_)_2_ is formed, with a coordination number of 8 and a distorted dodecahedral geometry. Due to its large ionic radius, thallium(III) prefers high coordination numbers (7 and 8) and forms stable structures even with ligands with a large number of donor atoms [67].

### 3.2. Complexes with f-Block Metals

Rare earth metals can form isostructural complexes with TPEN with the general formula [M(NO_3_)_2_(TPEN)][NO_3_]∙3H_2_O (M: La, Tb) and coordination number 10 [68]. By forming complexes in a 1:1 ratio, TPEN enables the effective separation of americium(III) from lanthanides from the aqueous phase to the organic phase (nitrobenzene), which is important in the waste disposal process. A single TPEN molecule binds much more strongly to the Am(III) ion than to Eu(III). This difference in binding strength is reflected in the distribution coefficient values (where D_M_ = [metal concentration in the organic phase]/[metal concentration in the aqueous phase]). At the same ligand concentration, the D_Am_ value is approximately two orders of magnitude higher than D_Eu_, allowing for the selective extraction of americium into the organic phase [69]. TPEN complexes with lanthanides exhibit specific thermodynamic and spectroscopic properties, and the stability of the formed compounds increases from lanthanum (La) to lutetium (Lu) with the decrease in ionic radius. The Y(III)/TPEN complex exhibits anomalous behavior; it is much less stable than its ionic radius would suggest (it behaves as an ion with a size similar to Ce(III)), which results from the lower degree of covalency of the metal–ligand bond compared with those of the lanthanides. In lanthanide complexes, the covalency is mainly due to charge transfer from the ligand to empty 5d orbitals and, to a smaller but noticeable extent, to 4f orbitals. In the case of yttrium, the share of 4d orbitals in the bond is smaller, resulting in a more ionic character of the bond [7]. Because of the much higher stability of complexes with Am(III) compared with those with Ln(III), TPEN and similar polypyridyl ligands are being investigated as extractants for the removal of americium from nuclear waste. TPEN can dissolve in ionic liquids and undergo protonation. This may allow lanthanides to be extracted from ionic liquids and the extracted metal ions to be released into an aqueous acid solution. Therefore, this ligand allows for the replacement of volatile, flammable, and toxic organic solvents with safer alternatives [70]. In high-level liquid waste, the total amount of lanthanides is much larger than that of actinides, which poses a significant challenge to the selectivity of extractants, which must precisely distinguish between almost chemically identical ions in an environment where one of them (lanthanide) dominates quantitatively and interferes with key technological processes [71]. The efficiency of separation of Am(III) ions from europium(III) using TPEN and its derivatives was also investigated using DFT calculations. It was presented that the bonds in americium complexes are slightly more covalent than in europium complexes, which favors their selective binding [72]. The stability constant of the [Am(TPEN)]^3+^ complex (logK = 6.69 ± 0.03 or 6.77 ± 0.01) is two orders of magnitude higher than that of [Sm(TPEN)]^3+^ (logK = 4.70 ± 0.02), so actinides interact more strongly with the “soft” nitrogen donors present in TPEN than lanthanides, which was also attributed to a slightly higher covalent contribution to the actinide bonds [73]. Hydrophobic TPEN derivatives can also be used to separate Am(III) from Ln(III), in which alkyl groups are introduced into the ligand skeleton [71,74] to reduce their water solubility. Theoretical calculations have shown that in the presence of Am^3+^/Eu^3+^ ions, TPEN exhibits selectivity towards Am^3+^ [14]. Separation of americium(III) from europium(III) is possible using a thermosensitive gel derived from TPEN, which acts as a cross-linking agent, allowing for the development of more ecological separation processes [75]. In the reaction of separating americium(III) from lanthanides(III), TPEN can also be used in combination with lipophilic carboxylic acids (decanoic acid or octanoic acid) in 1-octanol solution, and this system is safer for the environment because it replaces toxic nitrobenzene with 1-octanol and uses only completely flammable reagents, which eliminates the generation of secondary waste (as opposed to organophosphate extractants) [76]. TPEN is insoluble in nonpolar solvents (e.g., hexane and dodecane) but soluble in polar solvents such as nitrobenzene, alcohols, and chloroform [77] and is freely soluble in 1-octanol and nitrobenzene [63]. TPEN was used as a model ligand for the synthesis of its hydrophobic derivatives, which were also studied in the separation of smaller actinides from lanthanides with a view to their application in spent nuclear fuel reprocessing [77]. The role of soft donor ligands (including TPEN) in the selective separation of smaller actinides from lanthanides was also described in a review [78].

## 4. TPEN Analogues

**Scheme 10:**
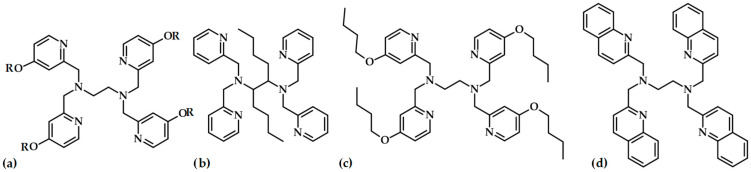
Structures of TPEN analogues (**a**) with fluoroalkyl groups F4TPEN R = CH_2_CF_2_CF_2_H, F5TPEN R = CH_2_CF_2_CF_3_, or F20TPEN R = CH_2_(CF_2_)_10_H, (**b**) TPDBEN, (**c**) TBPEN, and (**d**) TQEN.

The disadvantage of TPEN, which contributes to the inhibition of the metal extraction process, is its low resistance to environmental acidity and ease of protonation. Therefore, to improve the efficiency of metal ion extraction from acidic solutions, its derivatives modified with, for example, fluoroalkyl groups were synthesized (Scheme 10a). The synthesized structures were characterized by more efficient extraction of cadmium(II) and americium(III) ions into the “fluorous solvent” even at low pH (approx. 1–3). The obtained ligands also allowed for the selective separation of americium(III) from lanthanide ions, such as europium(III) [79], which may be useful in shortening the time of safe nuclear waste storage [79]. A review paper [80] was also devoted to the design and synthesis of various TPEN derivatives to optimize the processes of separating actinides from lanthanides, including in nuclear waste. TPEN has good selectivity for separating actinides from lanthanides, which is crucial to shortening the storage period of high-level radioactive waste. In the paper, the authors, in addition to modifying the ligand skeleton by introducing hydrophobic groups or polymerizable substituents, also discussed the use of TPEN-based polymer gels for selective metal capture [80]. TPEN derivatives that differ in the length of the diamine backbone have been used as cross-linking agents in reaction with *N*-isopropylacrylamide (NIPA) to form polymer gels. TPEN-NIPA gels exhibit reversible swelling and shrinking in water at a specific temperature, but the gels’ ability to trap cadmium ions decreases dramatically with the increase in temperature. TPEN ligand modification can be used to create polymer gels that allow for temperature-controlled extraction and release of metal ions, particularly for the selective capture of cadmium(II) from aqueous solutions [81]. TPEN and its hydrophobic analogues (TPDBEN–*N*,*N*,*N*′,*N*′-tetrakis(2-pyridylmethyl)dibutylethylenediamine [77] and TBPEN–*N*,*N*,*N*′,*N*′-tetrakis [4-(2-butyloxy)-2-pyridylmethyl]ethylenediamine [82]; Scheme 10b,c) were used in solvent extraction of perrhenate ions (ReO_4_^−^) as functional ligands capable of recognizing and removing hazardous and highly soluble ions. In the case of TPEN, the complexes formed are effectively extracted into chloroform at moderate pH; at low pH, the TPEN extraction efficiency decreases because the multiprotonated TPEN forms become too water-soluble. The use of hydrophobic TPEN analogues was desired to prevent the ligand from dissolving in the aqueous phase and allowed for a significant improvement in extraction in a strongly acidic medium [82].

TPEN loses its effectiveness in low-pH environments because it passes into the aqueous phase. The introduction of hydrophobic groups (e.g., butyloxy- groups in TBPEN) allows the ligand to be retained in the organic phase and achieves 100% extraction of cadmium(II) even at pH 1, while TPEN extracts less than 10% of this metal under the same conditions. TBPEN can be used for the recovery of valuable noble metals (Au, Pt, Pd, and Rh) and the removal of toxic heavy metals (Cd) from industrial waste [83]. The TPEN structure also served as a framework for the design of new advanced fluorescent sensors, e.g., for the TQEN ligand (*N*,*N*,*N*′,*N*′-tetrakis(2-quinolylmethyl)ethylenediamine; Scheme 10d), where the pyridine rings were replaced with quinoline groups [84]. Similarly, to build specific fluorescent sensors for zinc, the TPEN framework was used as a base for modifications consisting of replacing the pyridine rings in its structure with quinoline or isoquinoline groups. Modification of its arms (e.g., introduction of isoquinoline) was aimed at obtaining molecules that better distinguish Zn(II) from Cd(II) ions and exhibit less sensitivity to pH changes [85]. Modifications of the ligand molecule may find application in sensors capable of imaging mobile zinc inside cells (e.g., in rat PC-12 cells), which is important in the study of signaling processes and gene expression.

## 5. Summary and Outlook

TPEN is a neutral, *N*-hexadentate ligand capable of forming stable complexes with many metal ions of varying coordination numbers. Its high flexibility and ability to stabilize low oxidation states of metals make it one of the most frequently studied chelators in coordination chemistry and biomedicine. It is a highly specific chelator of Zn(II) ions, making it a tool for studying the intracellular transport of this ion and inducing functional deficiency of this element in cells (Table 2). TPEN can prevent hypoxia- and ischemia-induced neuronal death by inhibiting apoptosis and modulating zinc-dependent ion channels. This ligand can also be used to scavenge copper from SOD enzymes, leading to the generation of ROS, a reaction that could find applications in anticancer therapies. TPEN is also used in capillary electrophoresis to simultaneously detect multiple metal ions (including Fe, Cu, Ni, Pb, and Cd) in biological samples. Due to its selectivity in separating actinides from lanthanides in nuclear waste processing, TPEN may find applications in metal separation and the nuclear industry. It can also be used to produce intelligent polymers, and incorporating this ligand into thermosensitive gels enables temperature-dependent metal extraction, which could revolutionize the recovery of valuable or toxic metals from industrial wastewater.

**Scheme 11:**
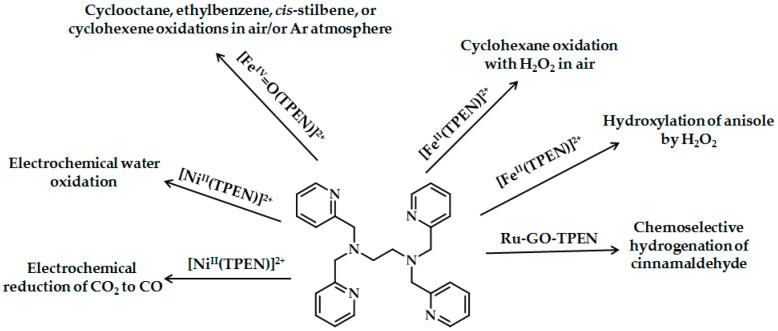
Reactions catalyzed by TPEN complexes [10,12,18,19,36,65].

TPEN complexes with various metal ions were investigated for their ability to catalyze different reactions (Scheme 11). Catalytic studies involving complexes based on TPEN or its derivatives often assume that the ligands remain fully bound to the metal center throughout the process.

However, this hypothesis has not yet been experimentally confirmed, creating a gap in the full understanding of the catalytic pathways. Furthermore, although relationships between the structure, spin state, and reactivity of the complexes have been demonstrated in the literature, there is very little reported experimental data directly supporting these claims. TPEN’s potential and challenges also lie in advanced nuclear waste separation and the development of intelligent materials and sensors, such as fluorescent ones. Unfortunately, TPEN’s high solubility in water and ease of protonation in acidic environments hinder its practical application in industrial metal extraction.

Therefore, it is necessary to design hydrophobic derivatives that exhibit higher acidity tolerance and improved solubility in organic solvents while maintaining chelating capacity under low-pH conditions. Furthermore, copolymerization of TPEN with NIPA allows for the formation of thermoreactive gels that alter metal extraction capacity with temperature. The development of TPEN-inspired zinc phototraps faces a challenge due to the low quantum yield of photolysis, which requires the development of entirely new ligand design strategies to enable their practical biological applications. Further research into the release mechanisms is therefore necessary to enable a precise study of zinc homeostasis in living systems without undesirable side effects.

Further development of research using TPEN is associated with several key challenges. TPEN is a potent chelator not only of zinc(II) but also of iron and copper ions, which, with prolonged exposure, can lead to depletion of essential metal pools in cells and induce apoptosis. Therefore, it is necessary to increase its specificity and develop safe systems for precise delivery to and release at target cells. The future of TPEN in medicine depends on the development of advanced carriers, such as nanoparticles, activated by external stimuli (ultrasound or light), which will minimize systemic side effects. TPEN can also be used to identify metal-binding proteins in the proteome. Chelation of zinc with TPEN is a promising approach to counteracting neuronal death after ischemic stroke. Future research could focus on developing targeted therapies that modulate glutamate signals and ion channels to protect, for example, the brain. This is a powerful tool for discovering new drug targets and mechanisms of metal-dependent diseases. TPEN complexes can combine multiple metal centers, opening the way to the creation of a variety of multifunctional molecular systems with unique chemical properties.

## Figures and Tables

**Table 2:** Biological activity and action of the ligand TPEN in cellular systems and organisms, involving interactions mainly with Zn(II) ions.

No.	Activity, Biological Effect	Research Material/Mechanism of Action	Ref.
1.	Regulation of stress granules	HT22 cell line/It inhibits the formation and promotes the disassembly of stress granules containing the TIA-1 protein by chelating Zn(II) ions released during oxidative stress.	[45]
2.	Inhibition of dopamine release	Rat nucleus accumbens (NAc)/It inhibits the ethanol-induced rise in dopamine levels by chelating zinc; it lowers basal dopamine levels.	[46]
3.	Blocking inflammatory signaling	Cultures of human proximal tubule cells/TPEN via zinc chelation inhibits MAPK kinase pathways (SAPK/JNK, p38, and ERK1/2) activated by the CD40 ligand; it abolishes the production of the cytokines IL-8 and MCP-1.	[47]
4.	Proteome destabilization	Cell lysate (K562)/It causes a decrease in the stability of zinc-binding proteins. TPEN chelates zinc ions, and the proteins lose their metal ligands, leading to a reduction in their structural stability.	[48]
5.	Intracellular diagnostics	Melanoma cells (A375)/It is used to verify the specificity of fluorescent zinc sensors by quenching their signal upon binding Zn^2+^ ions.	[49]
6.	Neuroprotection	Rat neurons/It inhibits cell death and apoptosis induced by glucose and oxygen deprivation; it regulates glutamate signaling (NR2B and GluR2 subunits) as well as potassium and sodium channels.	[50]
7.	Neuroprotection	Wild mice (C57BL/6J) and human retinal pigment epithelial cell line/It protects neurons by inhibiting inflammatory processes and pathways leading to programmed cell death. It promotes regenerative processes in damaged nerves. However, at higher concentrations, it may exhibit toxicity, leading to reduced viability of retinal epithelial cells.	[55]
8.	Reversal of resistance to apoptosis in monocytes from individuals with HIV	Monocytes isolated from patients with HIV/Zinc is a potent inhibitor of caspase-3 (a protease crucial to apoptosis); its removal using TPEN relieves the blockade of this signaling pathway and enables programmed cell death.	[53]
9.	Inhibition of snake venom enzymes	*Echis carinatus* venom, Swiss Albino mice, and blood from human volunteers. It counteracts the pro-coagulant effect of the venom and restores normal plasma clotting time. It neutralizes the toxic effects of the bite without interfering with the physiological, calcium-ion-dependent blood clotting process.	[54]

## Data Availability

No new data were created or analyzed in this study. Data sharing is not applicable to this article.
